# Multisite assessment of the impact of a prenatal testing educational App on patient knowledge and preparedness for prenatal testing decision making

**DOI:** 10.1007/s12687-022-00596-x

**Published:** 2022-06-10

**Authors:** Patricia Winters, Kirsten J. Curnow, Alexandra Benachi, Maria Mar Gil, Belen Santacruz, Miyuki Nishiyama, Fuyuki Hasegawa, Haruhiko Sago

**Affiliations:** 1 Illumina, Inc., San Diego, CA USA; 2 Obstetrics and Gynecology Department, Hôpital Antoine Béclère, AP-HP, Université Paris Saclay, Clamart, France; 3 Obstetrics and Gynecology Department, Hospital Universitario de Torrejón and School of Medicine, Universidad Francisco de Vitoria, Madrid, Spain; 4 Center for Maternal-Fetal, Neonatal and Reproductive Medicine, National Center for Child Health and Development, 2-10-1 Okura, Setagaya-ku, Tokyo, 157-8535 Japan

**Keywords:** Noninvasive prenatal testing, Surveys and questionnaires, Health knowledge, Attitudes, Decision making, Patient satisfaction

## Abstract

In this study, we wanted to assess the impact of the use of a patient educational app on patient knowledge about noninvasive prenatal testing (NIPT) and preparedness for prenatal screening decision-making. A randomized control study was carried out at three international sites between January 2019 and October 2020. Study participants completed a pre-consultation survey and post-consultation survey to assess knowledge, satisfaction, and preparedness for prenatal screening consultation. Providers completed a post-consultation survey. In the control arm, the pre-consultation survey was completed prior to consultation with their prenatal care provider. In the intervention arm, the pre-consultation survey was completed after using the app but prior to consultation with their prenatal care provider. Mean knowledge scores in the 203 participants using the app were significantly higher pre-consultation (*p* < 0.001) and post-consultation (*p* < 0.005) than those not using the app. Higher pre-consultation knowledge scores in the intervention group were observed at all sites. Most (86%) app users stated they were “Satisfied” or “Very Satisfied” with it as a tool. Providers rated the intervention group as more prepared than controls (*p* = 0.027); provider assessment of knowledge was not significantly different (*p* = 0.073). This study shows that clinical implementation of a patient educational app in a real-world setting was feasible, acceptable to pregnant people, and positively impacted patient knowledge.

**Supplementary Information:**

The online version contains supplementary material available at 10.1007/s12687-022-00596-x.

## Introduction

First introduced into clinical care in 2011, noninvasive prenatal testing (NIPT) for common aneuploidy screening has been widely adopted across the globe (Ravitsky et al. 2021). NIPT analyzes cell-free DNA in a pregnant person’s blood and has the highest sensitivity and specificity for aneuploidy screening of available prenatal screening tests (Gil et al. 2017). Globally, professional medical societies recognize NIPT as an appropriate screening option for pregnant patients and emphasize the importance of pre-test counseling prior to prenatal screening and the need for informed choice.

According to Marteau, “an informed choice is one that is based on relevant knowledge, consistent with the decision maker’s values and behaviorally implemented” (Marteau et al. 2001). Evidence has not yet supported the concern (Kater-Kuipers et al. 2018) that the ease of use with cell-free DNA screening will lead to “routinization” of testing and erode pregnant people’s informed choice (Cernat et al. 2019). Historically, counseling regarding prenatal screening and testing options has focused on ensuring that pregnant people have sufficient knowledge to make an informed decision. However, given concerns about limited time and resources available for pretest counseling, there is a trend toward the providers’ role shifting from primarily being that of information-giver to that of decision-making facilitator.

NIPT Insights is an app-based patient educational tool designed to aid prenatal care providers in the dissemination of the foundational knowledge required as a component of informed choice. By providing pregnant people with the information prior to consultation with their providers, incorporating the app into clinical care will enable the provider to use the limited face-to-face consultation time in a shared decision-making model facilitating a decision that is consistent with the pregnant person’s beliefs and values.

The main objective of this study was to evaluate the effectiveness of NIPT Insights as an adjunct to provider counseling regarding NIPT on patients’ preparedness for discussion with their providers about prenatal screening and testing options. Secondary objectives were to assess patient satisfaction with the app and impact of the app-based educational tool on time spent counseling about prenatal screening and testing options by the prenatal provider.

## Materials and methods

This was a randomized controlled trial implemented at three sites: An Ultrasound Unit in Madrid, Spain; a Fetal Medicine Unit with dedicated aneuploidy screening clinics in Clamart, France; and a Prenatal Genetics clinic in Tokyo, Japan. Pregnant people who were being offered NIPT as part of their routine care, and who were 18 years of age or older and spoke either English, French, Spanish, or Japanese, were eligible for participation. Participants were recruited between January 2019 through October 2020. Participants were randomized into one of two arms at the provider level. In the control arm, participants received routine care regarding prenatal screening and testing options, which varied across sites. Routine care in Spain included consultation with an obstetrician; in France, consultation with a dedicated midwife or genetic counselor; and in Japan, consultation with a genetic counselor. In the intervention arm, participants were provided access to the app-based patient educational tool in addition to routine care. Participants in the intervention group were provided with an iPad with access to NIPT Insights [https://apps.apple.com/us/app/nipt-insights/id1408704012; https://play.google.com/store/apps/details?id=eu.fiveminutes.illumina&hl=en_US&gl=US] for review in the waiting room prior to their consultation.

NIPT Insights was developed by Illumina, Inc. The content was written by three board-certified genetic counselors. Input was incorporated from maternal–fetal medicine specialists, patient advocacy groups, and focus groups of mothers of children with medical conditions from multiple countries throughout the world. The app was tested by women of reproductive age for input on design. NIPT Insights provides information about prenatal screening and testing options, with an emphasis on NIPT. It is available in multiple languages, with country-specific content. Features of the app include values assessment questions, ability to save topics of interest for further conversations, and the ability to email a summary of the participants’ app journey to themselves or their providers.

All study participants were asked to complete both a pre-consultation survey and post-consultation survey. The pre-consultation survey consisted of 25 questions, including 10 demographic questions and 15 knowledge-based questions (see Online Resource 1, Supplementary Table 1). The knowledge questions were modeled after the Maternal Serum Screening Knowledge Questionnaire (Goel et al. 1996) but updated to include NIPT. The post-consultation survey consisted of 24 questions for both groups, consisting of nine questions related to preparedness for the consultation and the 15 knowledge-based questions from the pre-consultation survey, with an additional six questions related to satisfaction with using the app for the intervention arm only (see Online Resource 1, Supplementary Table 2). Finally, each participant’s care provider was asked to complete a short provider survey consisting of five questions related to the length of time spent counseling about screening and testing options and subjective assessments of the participant’s preparedness for the consultation and knowledge level (see Online Resource 1, Supplementary Table 3).

The pre-consultation survey was completed after using the app-based tool but prior to consultation with their prenatal care provider in the intervention arm and prior to consultation with their prenatal care provider in the control arm. Participants in both arms completed the post-consultation survey following consultation with their prenatal care provider. The participants’ prenatal care provider completed the provider survey following the consultation with the participant. The surveys were conducted through SurveyMonkey on a device provided to the participants at their appointments. Unique participant codes were used to link each participant’s surveys. Participants that failed to complete both surveys were excluded from the analysis, regardless of whether the provider survey was completed for that participant.

### Statistical analyses

Knowledge scores and rating questions were calculated by summing the responses using − 2, − 1, 0, 1, 2 points corresponding to the five-point Likert scale answer. A value of 2 is assigned if the correct response was given with a “strongly” agree or disagree, and a value of 1 for correct response with an agree or disagree statement. Incorrect responses are assigned a value of − 2 and − 1, respectively, while a value of 0 is assigned for a neutral response. Given 15 knowledge questions, the potential range for knowledge scores was − 30 to 30. Knowledge and satisfaction scores for each participant and summary statistics including mean, standard deviation, and range were calculated. Comparisons between groups were analyzed by Chi-square for categorical variables, ANOVA for continuous variables between three or more groups, and *t* tests for continuous variables between two groups.

Power calculations were based upon a two-sided *t* test assuming equal variance using PASS 15, assuming a standard deviation of around 15% in knowledge scores and with a goal of being able to detect a 10% absolute difference in knowledge score (e.g., 60% in controls and 70% in intervention group) and suggested a minimum number of participants of 60 per arm (120 total). We over-recruited due to possible additional variance based on provider and geography.

## Results

**Fig. 1:**
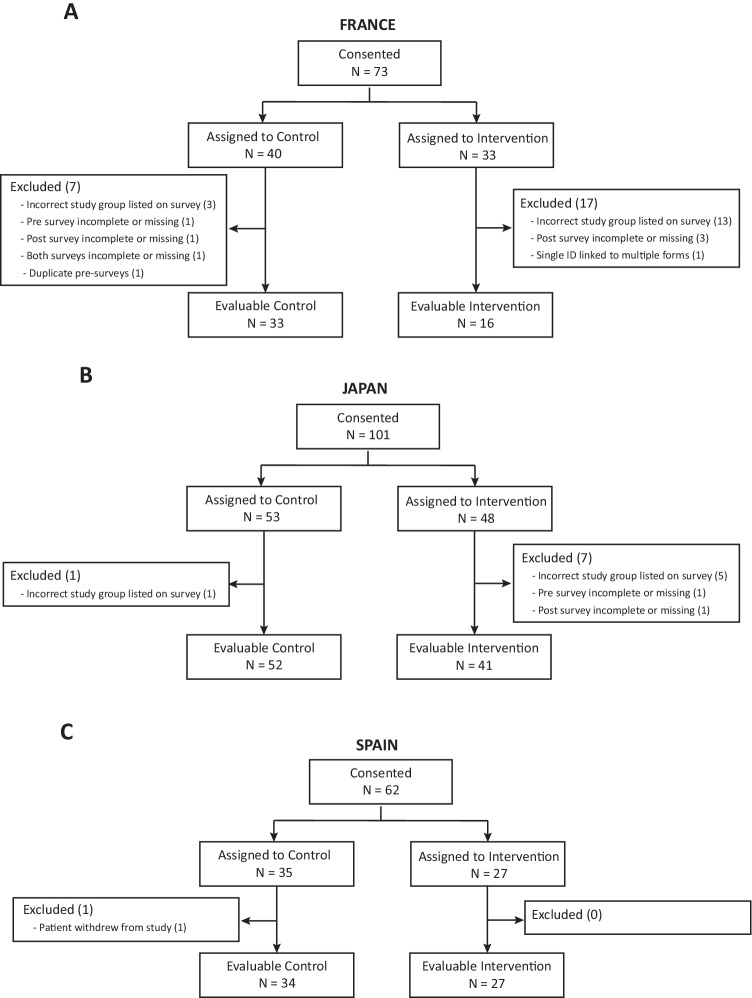
Study flow chart

A total of 236 people agreed to participate in the study across the three sites. Of these, 203 (86%) completed the pre-survey and the post-survey and were included in the analysis (Fig. 1). A total of 33 participants were excluded from the analysis: 22 listed the incorrect study group on their survey; eight had a missing or incomplete pre- and/or post-survey; one submitted more than one pre-survey; one had the same ID linked to multiple forms; and one patient withdrew from the study. Provider surveys were completed for 203 (100%) of the included participants. Table 1 shows the demographics of the study population.

**Table 1:** Demographics of the study population

Characteristic	France		Japan		Spain		Total	
	Control	Intervention	Control	Intervention	Control	Intervention	Control	Intervention
*N*	33	16	52	41	34	27	119	84
*Age*								
18–24 years	0 (0%)	0 (0%)	0 (0%)	0 (0%)	0 (0%)	1 (4%)	0(0%)	1(1%)
25–34 years	18 (55%)	9 (56%)	9 (17%)	7 (17%)	19 (56%)	16 (59%)	46 (39%)	32 (38%)
≥ 35 years	15 (45%)	7 (44%)	43 (83%)	34 (83%)	15 (44%)	10 (37%)	73 (61%)	51 (61%)
*Gestational age*								
< 10 weeks	0 (0%)	0 (0%)	0 (0%)	1 (2%)	0 (0%)	1 (4%)	0 (0%)	2 (2%)
10–14 weeks	19 (58%)	8 (50%)	44 (85%)	34 (83%)	34 (100%)	26 (96%)	97 (82%)	68 (81%)
15 weeks	14 (42%)	8 (50%)	8 (15%)	6 (15%)	0 (0%)	0 (0%)	22 (18%)	14 (17%)
*Number prior pregnancies*								
0	5 (15%)	2 (13%)	19 (37%)	14 (34%)	15 (44%)	12 (44%)	39 (33%)	28 (33%)
1	7 (21%)	4 (25%)	16 (31%)	10 (24%)	10 (29%)	7 (26%)	33 (28%)	21 (25%)
2 or more	21 (64%)	10 (63%)	17 (33%)	17 (41%)	9 (26%)	8(30%)	47 (39%)	35(42%)
*Number of children*								
0	8 (24%)	5 (31%)	34 (65%)	19 (46%)	17 (50%)	17 (63%)	59 (50%)	41 (49%)
1	13 (39%)	6 (38%)	13 (25%)	15 (37%)	10 (29%)	9 (33%)	36 (30%)	30 (36%)
2 or more	12 (36%)	5 (31%)	5 (10%)	7 (17%)	7 (21%)	1 (4%)	24 (20%)	13 (15%)
*Prior pregnancy with Down syndrome*								
Yes	3 (9%)	0 (0%)	3 (6%)	5 (12%)	0 (0%)	0 (0%)	6 (5%)	5 (6%)
No	30 (91%)	16 (100%)	49 (94%)	36 (88%)	34 (100%)	27 (100%)	113 (95%)	79 (94%)
*Highest level of education*								
No formal qualifications	2 (6%)	0 (0%)	0 (0%)	0 (0%)	0 (0%)	1 (4%)	2 (2%)	1 (1%)
High school and/or apprenticeship	3 (9%)	2 (13%)	7 (13%)	7 (17%)	11 (32%)	5 (19%)	21 (18%)	14 (17%)
Bachelors or equivalent	6 (18%)	2 (13%)	39 (75%)	31 (76%)	4 (12%)	6 (22%)	49 (41%)	39 (46%)
Graduate or higher	22 (67%)	12 (75%)	6 (12%)	3 (7%)	19 (56%)	15 (56%)	47 (39%)	30 (36%)
*Ethnicity*								
Black/African/Caribbean/N African	14 (42%)	5 (31%)	0 (0%)	0 (0%)	1 (3%)	0 (0%)	15 (13%)	5 (6%)
East Asian/South Asian/Japanese	0 (0%)	2 (13%)	52 (100%)	41 (100%)	0 (0%)	0 (0%)	52 (47%)	43 (51%)
Mixed/Multiple or Other	1 (3%)	1 (6%)	0 (0%)	0 (0%)	1 (3%)	1 (4%)	2 (2%)	2 (2%)
South American/Latin American	0 (0%)	0 (0%)	0 (0%)	0 (0%)	2 (6%)	2 (7%)	2 (2%)	2 (2%)
White	18 (55%)	8 (50%)	0 (0%)	0 (0%)	30 (88%)	22 (81%)	48 (40%)	30 (36%)
Prefer not to answer	0 (0%)	0 (0%)	0 (0%)	0 (0%)	0 (0%)	2 (7%)	0 (0%)	2 (2%)
*Primary language at home*								
French	31 (94%)	15 (94%)	0 (0%)	0 (0%)	0 (0%)	0 (0%)	31 (26%)	15 (18%)
Japanese	0 (0%)	0 (0%)	51 (98%)	41 (100%)	0 (0%)	0 (0%)	51 (43%)	41 (49%)
Spanish	0 (0%)	0 (0%)	0 (0%)	0 (0%)	32 (94%)	25 (93%)	32 (27%)	25 (30%)
English	1 (3%)	0 (0%)	1 (2%)	0 (0%)	0 (0%)	0 (0%)	2 (2%)	0 (0%)
Other	1 (3%)^a^	1 (6%)^b^	0 (0%)	0 (0%)	2 (6%)^c^	2 (7%)^¶d^	3 (3%)	3 (4%)
*Prior screening or testing*								
None	7 (21%)	2 (13%)	46 (88%)	38 (93%)	26 (76%)	22 (81%)	79 (66%)	62 (74%)
Amniocentesis	0 (0%)	0 (0%)	2 (4%)	0 (0%)	0 (0%)	0 (0%)	2 (2%)	0 (0%)
Serum screening	11 (33%)	6 (38%)	3 (6%)	0 (0%)	2 (6%)	1 (4%)	16 (13%)	7 (8%)
Ultrasound	5 (15%)	1 (6%)	0 (0%)	1 (2%)	2 (6%)	1 (4%)	7 (6%)	3 (4%)
Serum screening and	9 (27%)	6 (38%)	0 (0%)	1 (2%)	0 (0%)	0 (0%)	9 (8%)	7 (8%)
Ultrasound								
Other, Unsure, or Unanswered	1 (3%)	1 (6%)	1 (2%)	1 (2%)	4 (12%)	3 (11%)	6 (5%)	5 (6%)

Values are shown as *n* (%)

^a^Portuguese

^b^Arabic

^c^Arabic and German

^d^Arabic and Romanian

**Fig. 2:**
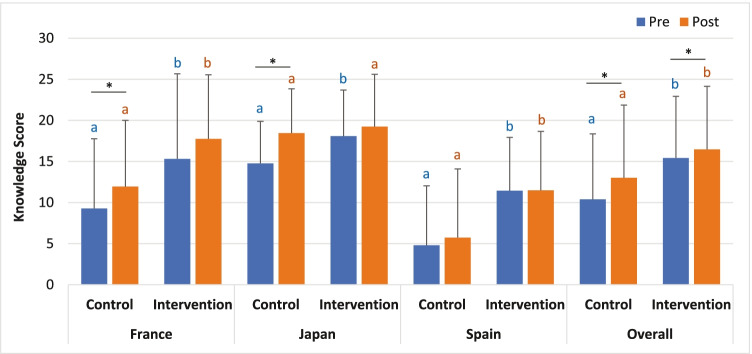
Impact of using an educational app on knowledge scores before and after physician visit. The control arm completed the pre-survey before their appointment with their health care provider. The intervention group completed the pre-knowledge survey after use of the educational app that provided information on prenatal testing but prior to their appointment with their health care provider. Both arms completed the post-survey immediately after their appointment with their health care provider. Different letters indicate a significant difference (*p* < 0.05) between pre- and post-knowledge scores within the control or intervention group

Overall, the mean knowledge scores in the participants using the app were significantly higher both pre-consultation (10.4 ± 8.0 vs. 15.4 ± 7.5; *p* < 0.001) and post-consultation (13.0 ± 9.9 vs. 16.5 ± 7.7; *p* < 0.005) than those in participants not using the app (Table 2 and Fig. 2). The mean knowledge score for the intervention group was 15 pre-consultation vs 16 post-consultation (*p* = 0.03) and for the control group was 10 pre-consultation and 13 (range, − 11–30; SD 8.8) post-consultation (*p* < 0.001). Although there was wide variation across the study sites, higher pre-consultation knowledge scores in participants using the app were observed at all sites (Table 2). The control groups at each site showed a larger improvement in knowledge scores from pre-survey to post-survey, suggesting that participants in the intervention group already had some of the knowledge typically discussed in consultation prior to meeting with their provider.

**Table 2:** Knowledge scores of study participants

	Pre-survey knowledge			Post-survey knowledge		
	Control Mean ± SD Range	Intervention Mean ± SD Range	*P* value	Control Mean ± SD Range	Intervention Mean ± SD Range	*P* value
Overall	10.4 ± 8.0 - 7 - 26	15.4 ± 7.5 - 4 -30	< 0.001	13.0 ± 8.8 - 11 - 30	16.5 ± 7.7 2 - 30	0.004
Spain	4.8 ± 7.2 - 6 - 26	11.4 ± 6.5 2 - 27	< 0.001	5.7 ± 8.4 - 11 - 24	11.5 ± 7.2 - 2 - 25	0.006
France	9.3 ± 8.5 7 - 24	15.3 ± 10.4 4 - 30	0.035	11.9 ± 8.1 - 2 - 25	17.8 ± 7.8 6 - 28	0.021
Japan	14.8 ± 5.1 5 - 26	18.1 ± 5.6 5 -29	0.004	18.4 ± 5.4 7 - 30	19.2 ± 6.4 5 - 30	0.513

**Fig. 3:**
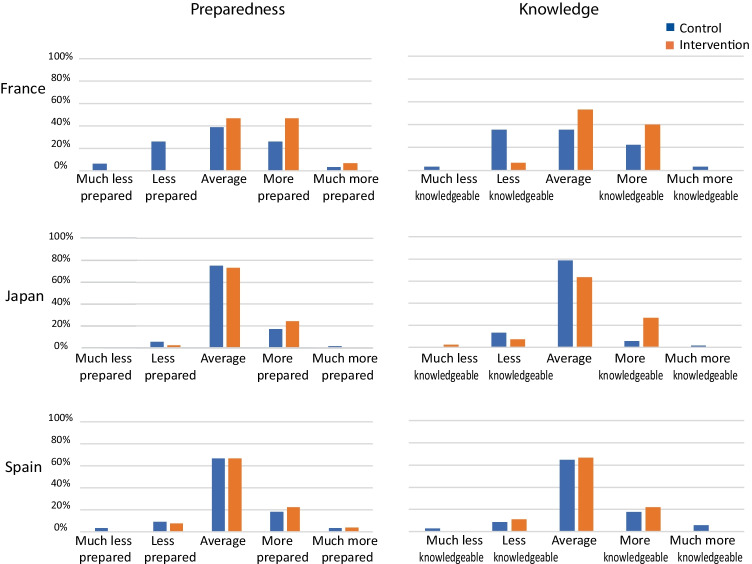
Provider rating of preparedness and knowledge

Most participants (137/203; 67%) indicated they felt they had enough information about prenatal screening and testing options prior to their appointment (see Online Resource 2, Supplementary Fig. 1). There was a wide variation in the amount of time and the resources used to gather information for the appointment (see Online Resource 2, Supplementary Fig. 2). The number of participants indicating they had enough information increased after their appointment (see Online Resource 2, Supplementary Fig. 1) to 179/203 (88%). In addition, 79% of participants stated they felt “Prepared” or “Very Prepared” to discuss screening and testing options with their provider (see Online Resource 2, Supplementary Fig. 1). Interestingly, when the participants’ providers were asked to subjectively rate the participants’ levels of knowledge and preparedness for the appointment (Fig. 3), participants in the intervention group had higher preparedness scores than the control group (*p* = 0.027); provider assessment of knowledge was not significantly different between the groups (*p* = 0.073). For provider assessment of patient preparedness and knowledge at a country level, the only significant finding was higher preparedness in France (*p* = 0.020). Participants in both groups overwhelmingly reported knowing the differences in various prenatal screening and testing options (see Online Resource 2, Supplementary Fig. 3).

**Fig. 4:**
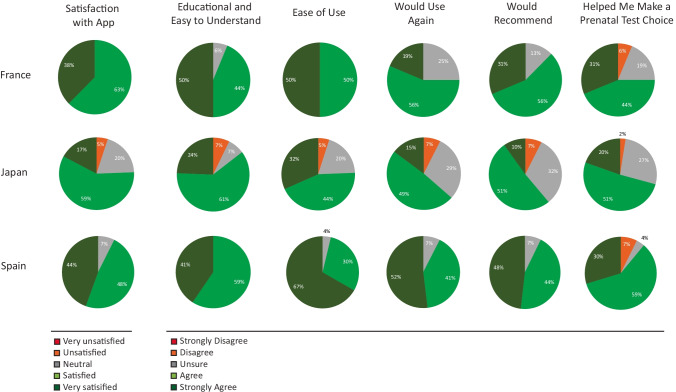
App satisfaction and ratings

Participants reported high satisfaction levels with the app (Fig. 4). Across sites the majority (86%) of participants using the app stated they were “Satisfied” or “Very Satisfied” with the patient educational app as a tool to learn more about prenatal screening and testing options. Most agreed the app was easy to use, easy to understand, and helped them make a prenatal test choice. In addition, most would use the app again and would recommend the app to others. Participants indicated multiple benefits to using the app and, to a lesser extent, some potential concerns with use of the app (see Online Resource 2, Supplementary Fig. 4).

On average, providers reported spending 16.3 min and 16.5 min discussing prenatal screening and testing options with participants in the control and intervention groups, respectively (*p* = 0.91). Of note, the time reported by providers varied between countries, with Spain typically reporting the lowest amount of time and Japan reporting the highest amount of time (see Online Resource 2, Supplementary Fig. 5). Within countries, there was no significant difference in the average time providers reported spending with patients between groups. Overall, most participants (92%) felt they had sufficient time to discuss prenatal screening and testing options with their providers. Interestingly, participants from France and Spain generally estimated they spent more time with the provider than reported by the provider.

## Discussion

We showed that the NIPT Insights educational app in conjunction with provider consultation increased patient knowledge about prenatal screening and testing options more than consultation alone. Participants with app access had higher knowledge scores pre- and post-consultation than those without app access. There was no difference in the time providers spent counseling about prenatal options between the two arms; however, providers did subjectively rate those with access to the app as more prepared for their consultation.

Sufficient knowledge is one component of informed choice. Prior to the introduction of NIPT, studies consistently demonstrated people had low-level understanding of prenatal aneuploidy screening (Gourounti and Sandall 2008; Jaques et al. 2004; Pop-Tudose et al. 2018; van den Berg et al. 2005), which persists in the NIPT era (Abousleiman et al. 2019). One-third of patients make uninformed choices regarding NIPT acceptance, predominantly due to insufficient knowledge (Beulen et al. 2016; Lewis et al. 2017). In one study of pregnant people with low-risk NIPT results in the current pregnancy, only 44% were able to correctly answer at least six of eight statements about NIPT and only 10% correctly answered all eight (Piechan et al. 2016). This effect is likely magnified in those with lower educational levels and lower health literacy and numeracy, who have been shown to have lower knowledge scores for prenatal genetic testing (Cho et al. 2007). The high educational status of participants in this study precludes examination of the potential effectiveness of an app-based tool in people with lower educational levels. More recently, studies have shown that pregnant people in France (Wehbe et al. 2020) and the USA (Palomaki et al. 2017) are more knowledgeable about aneuploidy screening and NIPT than previously suggested.

Professional societies emphasize the need for pre-test counseling to facilitate informed decision-making. With expanding use and testing options of NIPT, there is a growing need to explore alternative counseling approaches. We demonstrated that an app-based educational tool increased knowledge. Other studies investigating alternative service delivery models show mixed results. Patient knowledge and self-reported understanding was shown to be positively impacted by use of educational videos (de Leeuw et al. 2019; Mulla et al. 2018). A randomized controlled trial in the USA found that using a computerized interactive decision support guide significantly increased informed choice of people considering amniocentesis (Kuppermann et al. 2014). Similarly, another US-based randomized control trial showed people using interactive technology in addition to standard counseling for prenatal screening demonstrated better knowledge than people receiving provider counseling only; this was consistent across diverse educational, health literacy, and electronic literacy backgrounds, suggesting that digital tools may be widely applicable (Yee et al. 2014). A similar trial in the Netherlands found a significant increase in informed reproductive decision-making associated with use of a web-based multimedia decision aid (Beulen et al. 2016). Carlson et al. demonstrated that knowledge scores in people using a computerized decision aid were not inferior to those of people having genetic counseling (Carlson et al. 2019). Conversely, Skjoth et al. failed to find a significant impact on informed choice with use of an interactive website (Skjøth et al. 2015) and a study in Thailand found that computer-assisted instruction was less effective in improving patient knowledge scores than was individual counseling in combination with a self-read leaflet in patients considering amniocentesis (Hanprasertpong et al. 2013).

Prenatal health care providers acknowledge a lack of time to adequately counsel pregnant patients about prenatal screening options (Gammon et al. 2016). This was echoed by findings of a meta-synthesis of patients’ experiences with NIPT, in which some patients felt that consultations were too short to address their questions and concerns (Cernat et al. 2019), probably influenced by background characteristics, experience, attitudes, and knowledge (Nishiyama et al. 2021). Patients described feeling overwhelmed by the amount of information and the limited time in which to process it (Cernat et al. 2019). In a survey of US-based obstetrical providers, the average time spent on pre-test discussion with the patient was 6 min (range, 2.5–15 min) (Palomaki et al. 2017). Here, the time providers reported spending with patients varied between countries. Most participants indicated they had enough information and sufficient time with their provider to discuss testing options. App use did not appear to impact the amount of time spent in consultation with a provider but may have positively impacted participants’ preparedness for this discussion, as assessed by their providers.

Here, 86% of intervention participants reported satisfaction with the app. Previous studies suggested that many pregnant people considering NIPT prefer getting information a few days before testing (Laberge et al. 2019; Lewis et al. 2014), involving partners in the decision-making process (Laberge et al. 2019; Portocarrero et al. 2017), and having information accessible at home (Portocarrero et al. 2017). This was reflected in app benefits reported by participants of this study. However, patients also strongly value consultation with their providers (Laberge et al. 2019), which was echoed by our findings with 5% of participants preferring only a provider consultation.

Strengths of this study include the randomized design and inclusion of geographically diverse regions, demonstrating the intervention effectiveness in different settings. Limitations of the study include differing routine care at each site, which may have impacted patient knowledge as measured in the post-survey. In addition, the baseline knowledge was not assessed in the intervention group. Given the high educational level of participants, we could not assess the impact of an app-based intervention in people with lower health literacy. In addition, participants were provided with access to NIPT Insights at the time of their testing appointment, rather than prior to the appointment as we would recommend for routine clinical use.

As NIPT use expands into routine clinical care for all pregnant people and in complexity with screening for a growing number of conditions, educational tools to facilitate pre-test counseling are needed. Educational tools, such as NIPT Insights, can provide the foundational information necessary to prepare pregnant people for consultation with their providers. An app-based approach addresses many of the preferences reported by pregnant people regarding pre-test counseling by providing information before a testing decision needs to be made and enabling them to reflect on the information in their home with their partner or other support persons. Importantly, equipping pregnant people with information prior to their consultation allows health care providers to utilize their limited consultation time to address specific questions and explore a patient’s values and beliefs, facilitating informed choice through shared decision-making. This study shows that clinical implementation of a patient educational app in a real-world setting was feasible, acceptable to pregnant people, and positively impacted patient knowledge.

## Supplementary Information

Below is the link to the electronic supplementary material.

Supplementary file1 (PDF 208 KB)

Supplementary file2 (DOCX 1.15 MB)

## Data Availability

The data that support the findings of this study are available from the corresponding author upon reasonable request.
