# Supplementary material for: Multisite assessment of the impact of a prenatal testing educational App on patient knowledge and preparedness for prenatal testing decision making
Source: J Community Genet. 2022 Jun 10;13(4):435–44. doi: 10.1007/s12687-022-00596-x (PMC9314500; doi:10.1007/s12687-022-00596-x)
Supplement: Supplementary file 1 — Supplementary file1 (PDF 208 KB) [file 12687_2022_596_MOESM1_ESM.pdf]

Article Title: Multisite assessment of the impact of a prenatal testing educational App on patient knowledge and preparedness for prenatal testing decision making

Journal Name: Journal of Community Genetics

Author Names: Patricia Winters, Kirsten J Curnow, Alexandra Benachi, Maria Mar Gil, Belen Santacruz, Miyuki Nishiyama, Fuyuki Hasegawa, Haruhiko Sago

Corresponding Author: Patricia Winters; Illumina, Inc. San Diego, CA, USA; pdwinters@illumina.com

Supplementary Table 1. Pre Survey

The information we learn during this study will help us better teach other pregnant women about prenatal screening and testing options. First, we would like you to tell us a little about yourself.

Thank you for participating in this important research.

1. Please enter your participant code:

2. How old are you?

- 18-24 years
- 25-29 years
- 30-34 years
- 35-39 years
- 40-44 years
- 45-49 years
- 50 years or older

3. What is your ethnicity?

- White
- South American / Latin American
- South Asian / Japanese
- East Asian
- Black / African / Caribbean / North African
- Other ethnic group
- Mixed/Multiple ethnic groups
- Prefer not to answer

4. What is the highest level of education you have completed?

- No formal qualifications
- High school education and/or apprenticeships
- Bachelors degree or equivalent
- Graduate degree and higher qualifications

5. How far along are you in this pregnancy?

- <10 weeks
- 10-14 weeks
- 15-20 weeks
- 21-24 weeks
-
- 25-29 weeks
- 30 or more weeks
- Unsure

6. Other than this pregnancy, how many pregnancies have you had?

- 0
- 1
- 2
- 3 or more

7. Other than this pregnancy, how many children have you had?

- 0
- 1
- 2
- 3 or more

8. What is the language you use most often at home?

- English
- Spanish
- French
- Italian
- German
- Polish
- Welsh
- Romanian
- Arabic
- Indian and Pakistani languages (Urdu, Bengali, Gujarati, and
- Punjabi)
- Chinese
- Japanese
- Other (please specify)

9. Have you had a pregnancy diagnosed with Down syndrome (trisomy 21)?

- Yes
- No
- Prefer not to answer

10. Please check any screening or testing for Down syndrome you have had *in this pregnancy* prior to your appointment today?

- Ultrasound
- Serum screening (eg, first trimester screening [combined test])
- Chorionic Villus Sampling (CVS)
- Amniocentesis
- None
- Not Sure
- Other (please specify)

Now we would like you to read a set of statements, and tell us how close each statement comes to your own view.

	Strongly Agree	Agree	Unsure	Disagree	Strongly Disagree
11. If a noninvasive prenatal test (NIPT) result is positive, further tests are needed to tell if the pregnancy has one of the conditions.					
12. Chorionic villus sampling (CVS) and amniocentesis can tell with more certainty than noninvasive prenatal testing (NIPT) whether a pregnancy has Down syndrome (trisomy 21).					
13. If noninvasive prenatal testing (NIPT) results are positive, this means that the pregnancy has Down syndrome (trisomy 21).					
14. Women who have a negative noninvasive prenatal test (NIPT) can be sure that they will have a healthy baby.					
15. Screening tests tell you for sure whether your pregnancy has Down syndrome.					
16. All pregnant women are obligated to have prenatal screening for chromosomal conditions.					
17. Noninvasive prenatal testing (NIPT) can be used to detect every kind of fetal health condition.					
18. Chorionic villus sampling (CVS) and amniocentesis are associated with a risk for complications, including miscarriage.					
19. Noninvasive prenatal testing (NIPT) consists of a blood test on the mother.					
20. The chance of having a baby with Down syndrome gets higher as women get older.					
21. Children with Down syndrome (trisomy 21) have intellectual disability.					
22. Amniocentesis involves taking blood from a pregnant woman's arm.					
23. First trimester screening (FTS) is more likely than noninvasive prenatal testing (NIPT) to give a false positive result.					

24. A normal noninvasive prenatal test (NIPT) result means there is no longer a chance for the pregnancy to have Down syndrome.					

Supplementary Table 2. Post Survey

1. Please enter your participant study number:

2. How would you rate the amount of information about prenatal screening and testing options you had before coming to today's appointment?
- Enough information
- Unsure
- Not enough information

3. What resources did you use before today's appointment to learn about prenatal screening and testing options?
- Consultation with health specialist
- Websites
- Pamphlets
- TV, radio, or podcast
- Magazines or newspapers
- Friends and/or family
- Previous pregnancy
- School or work
- Other (please specify)

4. Please tell us which websites you used, if any.

5. How much time did you spend before today's appointment looking for information about prenatal screening and testing options?
- None
- Less than 10 minutes
- 10 to 30 minutes
- 30 to 60 minutes
- More than 1 hour

6. After today's visit, how would you rate the amount of information you now have about prenatal screening and testing options?
- Enough information
- Unsure
- Not enough information

7. At today's visit, what resources did you use to learn more about prenatal screening and testing options?
- Consultation with health specialist
- App and consultation with health specialist

- 8. How prepared did you feel to discuss your prenatal testing options with your health specialist today?
  Very prepared
- Prepared
- Unsure
- Unprepared
- Very unprepared

9. How much time did you spend with your health specialist today discussing prenatal screening and testing options?
- Less than 5 min
- 5-9 min
- 10-19 min
- 20-29 min
- 30 min or more

10. Was this time with your health specialist today discussing prenatal screening and testing options sufficient?
- I would have preferred more time with my health specialist
- I had sufficient time
- Neutral
- I would have preferred less time with my health specialist

11. At this time, how well do you know the differences in various prenatal screening and testing options:

	Strongly Agree	Agree	Unsure	Disagree	Strongly Disagree
I know the risks of different options					
I know the benefits of different options					
I know when during pregnancy these options are available to me					
I feel like I have enough information to make a choice about what type of screening or testing I want (if any)					

12. How satisfied are you with the patient educational app to learn more about prenatal screening and testing options?

- Very satisfied
- Satisfied
- Neutral
- Unsatisfied
- Very unsatisfied

13. How would you rate the following statements with regard to the app?

	Strongly Agree	Agree	Unsure	Disagree	Strongly Disagree
The information in the educational app was easy to understand.					
The app was easy to use.					
I would use this app again.					
I would recommend this app to family and friends.					
The information in the app helped me make a choice about which prenatal screening or testing option I chose.					

14. What language did you view the app in?

- English
- French
- Spanish
- Japanese

15. What are some good things about using the app? Please select all that apply.

- Ability to review the material at my own pace
- Convenience
- The ability to use at home before my appointment (Not an
- option in this study, but it is a planned future feature)
- Ability to use the app with my partner/other support person
- Ability to record questions for my provider
- Provided sufficient information on the different types of
- prenatal screening and testing options
- Provided sufficient information on the risks and benefits for
- each prenatal screen and test option
- The app provided fair and balanced information
- Other (please specify)

16. What are some things that concern you about using the app? Please select all that apply.

- Privacy
- Limited cellular data to access/download app on my personal
- device
- Connectivity issues
- Too much time
- Too complicated
- Not enough information
- Too much information
- Lack of access to smart phone or iPad
- Prefer to just discuss with provider
- Other (please specify)

17. Please provide any additional comments or suggestions related to your experience with the app.

Now we would like you to read a set of statements, and tell us how close each statement comes to your own view

	**Strongly Agree**	**Agree**	**Unsure**	**Disagree**	**Strongly Disagree**
11. If a noninvasive prenatal test (NIPT) result is positive, further tests are needed to tell if the pregnancy has one of the conditions.					
12. Chorionic villus sampling (CVS) and amniocentesis can tell with more certainty than noninvasive prenatal testing (NIPT) whether a pregnancy has Down syndrome (trisomy 21).					
13. If noninvasive prenatal testing (NIPT) results are positive, this means that the pregnancy has Down syndrome (trisomy 21).					
14. Women who have a negative noninvasive prenatal test (NIPT) can be sure that they will have a healthy baby.					
15. Screening tests tell you for sure whether your pregnancy has Down syndrome.					
16. All pregnant women are obligated to have prenatal screening for chromosomal conditions.					
17. Noninvasive prenatal testing (NIPT) can be used to detect every kind of fetal health condition.					
18. Chorionic villus sampling (CVS) and amniocentesis are associated with a risk for complications, including miscarriage.					
19. Noninvasive prenatal testing (NIPT) consists of a blood test on the mother.					
20. The chance of having a baby with Down syndrome gets higher as women get older.					
21. Children with Down syndrome (trisomy 21) have intellectual disability.					
22. Amniocentesis involves taking blood from a pregnant woman's arm.					
23. First trimester screening (FTS) is more likely than noninvasive prenatal testing (NIPT) to give a false positive result.					
24. A normal noninvasive prenatal test (NIPT) result means there is no longer a chance for the pregnancy to have Down syndrome.					

Supplementary Table 3. Provider Survey

1. Please enter the participant's study number.

2. How much time did you spend reviewing and discussing prenatal screening and testing options with this patient? Please exclude time patients spent completing paperwork and time spent discussing other matters.

-
- <5 minutes
- 5-9 minutes
- 10-19 minutes
- 20-29 minutes
- ≥30 minutes

3. Please enter a numerical value of your estimated time (in minutes).

4. In your opinion, please rate this patient's preparedness to discuss prenatal screening and testing options

- compared to other patients in your clinic.
  Much more prepared
- More prepared
- Average
- Less prepared
- Much less prepared

5. In your opinion, please rate this patient's knowledge about prenatal screening and testing options at the beginning of your discussion compared to other patients in your clinic.

- Much more knowledgeable
- More knowledgeable
- Average
- Less knowledgeable
- Much less knowledgeable
