# Supplementary material for: Multisite assessment of the impact of a prenatal testing educational App on patient knowledge and preparedness for prenatal testing decision making
Source: J Community Genet. 2022 Jun 10;13(4):435–44. doi: 10.1007/s12687-022-00596-x (PMC9314500; doi:10.1007/s12687-022-00596-x)
Supplement: Supplementary file 2 — Supplementary file2 (DOCX 1.15 MB) [file 12687_2022_596_MOESM2_ESM.docx]

# Article Title: Multisite assessment of the impact of a prenatal testing educational App on patient knowledge and preparedness for prenatal testing decision making

**Journal Name:** Journal of Community Genetics

**Author Names:** Patricia Winters, Kirsten J Curnow, Alexandra Benachi, Maria Mar Gil, Belen Santacruz, Miyuki Nishiyama, Fuyuki Hasegawa, Haruhiko Sago

**Corresponding Author:** Patricia Winters; Illumina, Inc. San Diego, CA, USA; [pdwinters@illumina.com](mailto:pdwinters@illumina.com)

**Supplementary Figure 1.** Information participants felt they had before and after today’s appointment and patient reported level of preparedness after today’s visit.

**Supplementary Figure 2.** Time and resources used before today’s visit.

**Supplementary Figure 3.** Patient reporting of their understanding of prenatal testing options.

**Supplementary Figure 4.** App likes and dislikes.

Participants in the Intervention group were asked “What are some good things about using the app?” (A) and “What are some things that concern you about using the app?” (B) and asked to select all that applied.

*Planned future feature.

**Supplementary Figure 5.** Provider and patient assessment of time with provider.
